# The complete chloroplast genome of weedy rice *Oryza sativa* f. *spontanea*

**DOI:** 10.1080/23802359.2021.1977197

**Published:** 2021-09-22

**Authors:** Chenfeng Dong, Yiyu Hu, Chuyu Ye, Longjiang Fan, Longbiao Guo

**Affiliations:** a Institute of Crop Sciences, College of Agriculture and Biotechnology, Zhejiang University, Hangzhou, China; b State Key Laboratory of Rice Biology, China National Rice Research Institute, Hangzhou, China

**Keywords:** Chloroplast genome, *Oryza sativa* f. *spontanea*, phylogenetic analysis

## Abstract

The emergence of weedy rice (*Oryza sativa* f. *spontanea*) has been considered as a serious global agricultural problem in recent decades. To better understand its speciation, here we assembled the complete chloroplast genome of *O. sativa* f. *spontanea* with the length of 134,502 bp. The assembly contains a large single-copy (LSC, 80,549 bp), a small single-copy (SSC, 12,347 bp) and a pair of inverted repeats (IRa and IRb, 20,803 bp each). A total of 132 unique genes were annotated, including 82 protein-coding genes, 42 tRNA genes and eight rRNA genes. Phylogenetic analysis showed that *O. sativa* f. *spontanea* (*indica* type) appears closely related to cultivated *indica* rice rather than wild rice, supporting the hypothesis that weedy rice originated from cultivated rice.

Weedy rice (*Oryza sativa* f. *spontanea*) refers to the unwanted *Oryza* plants in paddy fields that appear with some undesired agronomic traits, such as high degrees of seed shattering and seed dormancy, dramatically distinguished from cultivated rice (Qiu et al. 2020). At present, weedy rice has become one of the most common and noxious weeds in global paddy fields and has caused serious agricultural problems, as it could reduce the crop yields by greater than 80% without artificial weeding (Estorninos et al. 2005; Oerke 2006; Ziska et al. 2015). As its genomic background is similar to cultivated rice, it is difficult to eliminate weedy rice by applying regular herbicides (Nadir et al. 2017). Chloroplast genomes provide useful information to species identification and evolutionary studies. No chloroplast genomes of *O. sativa* f. *spontanea* have been available. Thus, here we assembled the complete chloroplast genome of *O. sativa* f. *spontanea* (*indica* type).

The sample of *O. sativa* f. *spontanea* (*indica* type) used in this study was collected in Changxing County, Zhejiang Province, China (30°55′17.7″N, 119°51′58.9″E) and deposited in the Herbarium of Zhejiang University (HZU, http://sweetgum.nybg.org/science/ih) with the accession number HZU60244003. The whole-genome high-throughput sequencing data was generated throught our previous study (Qiu et al. 2020). The raw reads were first filtered into clean data using NGSQCtoolkit v2.3 (Patel and Jain 2012). Using the cultivated *indica* rice (*Oryza sativa*) complete chloroplast genome (GenBank accession number NC_031333.1) as a reference, NOVOPlasty v3.6 (Dierckxsens et al. 2017) was used in the *de novo* assembly. Genome annotation was performed by the GeSeq online (Tillich et al. 2017). The assembled genome sequences and annotation information have been deposited in GenBank under the accession number LC642244.

The total length of *O. sativa* f. *spontanea* (*indica* type) chloroplast genome is 134,502 bp, which displays the typical quadripartite structure of most angiosperm chloroplast genomes, including the large single-copy (LSC, 80,549 bp), the small single-copy (SSC, 12,347 bp) and a pair of inverted repeats (IRa and IRb, 20,803 bp each). The GC contents of the LSC and SSC, IR regions are 37.1%, 33.3% and 44.3%, respectively. A total of 132 unique genes were annotated, including 82 protein-coding genes, 42 tRNA genes and eight rRNA genes. 21 genes were duplicated in the IR regions.

To help clarify understand the phylogenetic relationship between *O. sativa* f. *spontanea* (*indica* type) and other *Oryza* species, the chloroplast genome sequences of ten *Oryza* species and *Leersia perrieri* were downloaded from GenBank and used for phylogeny construction. We first performed sequence alignment using MAFFT v7.310 (Katoh et al. 2002) with the parameter ‘–auto –reorder –phylipout.’ IQ-tree v1.6.12 was then used to construct the phylogenetic tree using *L. perrieri* as an outgroup with the recommended parameter ‘-m MFP -bb 1000 -bnni’ (Nguyen et al. 2015). Finally, the tree was illustrated and modified by iTOL (Letunic and Bork 2019). The phylogenetic result showed that *O. sativa* f. *spontanea* (*indica* type) was genetically close to cultivated rice *O. sativa* (*indica* type) rather than *O. nivara* or other wild rice (Figure 1), which supported the hypothesis that *O. sativa* f. *spontanea* originated from cultivated rice (Qiu et al. 2017).

**Figure 1.**
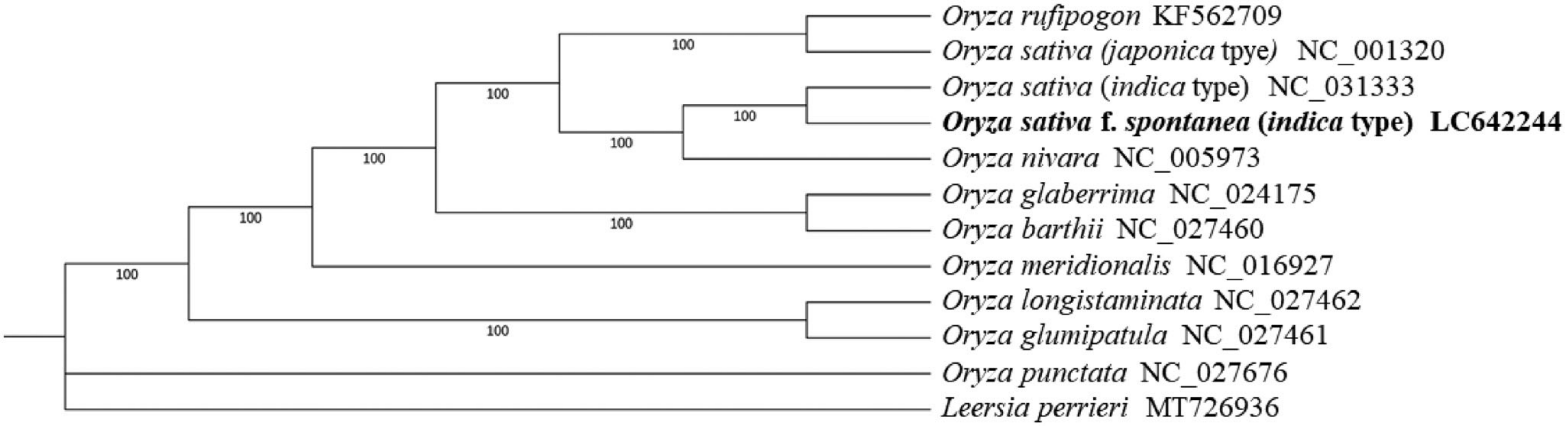
Maximum-likelihood phylogenetic tree of 12 species (varieties) based on complete chloroplast genomes. Bootstrap support value from 1000 replicates is shown on each node.

## Data Availability

The genome sequence data of this study is available in GenBank of NCBI at (https://www.ncbi.nlm.nih.gov) under the accession number LC642244. The associated BioProject, SRA, and BioSample numbers are PRJNA606132, SRX7710350, and SAMN14085936 respectively.
